# Supplementary material for: Content-rich biological network constructed by mining PubMed abstracts
Source: BMC Bioinformatics. 2004 Oct 8;5:147. doi: 10.1186/1471-2105-5-147 (PMC528731; doi:10.1186/1471-2105-5-147)
Supplement: Additional File 2 — The original results of the above study (non-essential files are deleted to keep the file size under the limit set by BMC bioinformatics). [file 1471-2105-5-147-S2.bz2 › chilibotAdditionalFile2/dip50/755ID8631671E18141/html/EXBB_EXBD.html]

 


 **EXBB** and **EXBD** 
  
Found 56 abstracts in PubMed, retrieved 50.  
 

 What does Google say? 
 PDF only 
| .edu only 

---

**Interactive relationship** (e.g. stimulation, inhibition, etc)

**Neutral relationship**- The aim of this review is to describe an in vivo assay of the interactions taking place in the Tol Pal or TonB  **ExbB**   **ExbD**  envelope complexes in the periplasm of Escherichia coli and between them and colicins or g3p protein of filamentous bacteriophages.  Ref: 12423784 Biochimie< MedlineTA>Biochimie,
- Plasmid encoded Hi  **exbB**  and  **exbD**  partially complemented an E. coli  **exbB**   **exbD**  mutation.  Ref: 7828935 Gene, 1995
- Interactions in the TonB dependent energy transduction complex  **ExbB**  and  **ExbD**  form homomultimers.  Ref: 9811664 J Bacteriol, 1998
- Energy for active transport is obtained from the proton motive force of the inner membrane through physical interaction with TonB  **ExbB**   **ExbD** , an inner membrane complex.  Ref: 9886293 Nat Struct Biol, 1999
- The phenotypes of  **exbB**   **exbD**  strains were essentially indistinguishable from the phenotypes of  **exbB**   **exbD**  strains.  Ref: 7642501 J Bacteriol, 1995
- **ExbB**  TolR and  **ExbD**  TolQ mixtures showed little if any complementation of  **exbB**   **exbD**  and tolQ tolR mutants indicating coevolution of  **ExbB**  with  **ExbD**  and TolQ with TolR.  Ref: 8316079 Mol Microbiol, 1993
- The recent observation that  **ExbB**  exists in 3.5 fold molar excess relative to the molarity of  **ExbD**  in E. coli suggests the possibility of two types of complexes, those containing both  **ExbB**  and  **ExbD**  and those containing only  **ExbB** .  Ref: 12193634 J Bacteriol, 2002
- both were eluted with the  **ExbB**  labeled with 6 histidine residues, demonstrating interaction of  **ExbB**  with  **ExbD**  and TonB.  Ref: 8631671 J Bacteriol, 1996
- Together, these results support an alternative model where all of the  **ExbB**  in the cell occurs in complex with all of the  **ExbD**  in the cell.  Ref: 12193634 J Bacteriol, 2002
- The membrane topologies of TolQ and TolR are similar to those proposed for  **ExbB**  and  **ExbD** , respectively, which is consistent with the partial functional substitution between  **ExbB**  and TolQ and between  **ExbD**  and TolR.  Ref: 8331075 J Bacteriol, 1993
- The deduced Hi  **ExbB**  and  **ExbD**  proteins possessed 27 and 28% amino acid identity 56 and 58% relatedness with the Escherichia coli  **ExbB**  and  **ExbD**  proteins, respectively.  Ref: 7828935 Gene, 1995
- In this paper we show that  **ExbD**  protein, encoded by an adjacent gene in the exb cluster at 65, was also required for TonB dependent energy transduction and, like  **ExbB** , was required for the stability of TonB.  Ref: 7642501 J Bacteriol, 1995

**Non-interactive relationship** (e.g. studied together, co-existance, homology, etc.)

- It belongs to the group B colicins the uptake of which through the outer membrane depends on the TonB,  **ExbB**  and  **ExbD**  proteins.  Ref: 8341256 Mol Gen Genet, 1993
- **ExbB**  and  **ExbD** , despite being encoded from the same operon, were not equimolar, being present at 2463 522 and 741 105 copies respectively.  Ref: 11967085 Mol Microbiol, 2002
- and two cytoplasmic membrane proteins with less defined roles,  **ExbB**  and  **ExbD** .  Ref: 11967085 Mol Microbiol, 2002
- Cloning and functional characterization of Neisseria gonorrhoeae tonB,  **exbB**  and  **exbD**  genes.  Ref: 9140974 Mol Microbiol, 1997
- Transcription initiation of the fec transport genes required the Ton system, consisting of TonB,  **ExbB** , and  **ExbD** , and was inhibited by carbonylcyanide m chlorophenylhydrazone CCCP and dinitrophenol DNP, which dissipate the electrochemical potential of the cytoplasmic membrane.  Ref: 9044267 Mol Microbiol, 1997
- Activity of  **ExbB**   **ExbD**  via TolA was higher in cells lacking TonB, and activity of TolQ TolR via TonB was increased when TolA was missing.  Ref: 8316079 Mol Microbiol, 1993
- These data further support the concept that TonB,  **ExbB** , and  **ExbD**  form a complex in which the energized conformation of TonB opens the channels in the outer membrane receptor proteins.  Ref: 8631671 J Bacteriol, 1996
- These predicted protein sequences exhibited 32 38% and 24 36% identity, respectively, with  **ExbB**  and  **ExbD**  proteins of three other bacteria.  Ref: 9140974 Mol Microbiol, 1997
- A comparison of the DNA flanking the  **exbB**   **exbD**  tonB locus in P. haemolytica A1 and H. influenzae showed that the flanking regions are completely different between the two organisms.  Ref: 9074497 Gene, 1997
- Sequence homology and mutual functional substitution of  **ExbB**   **ExbD**  and TolQ TolR suggest the evolution of the two import systems from a single import system.  Ref: 8316079 Mol Microbiol, 1993
- The cytoplasmic membrane proteins  **ExbB**  and  **ExbD**  support TonB dependent active transport of iron siderophores and vitamin B12 across the essentially unenergized outer membrane of Escherichia coli.  Ref: 9811664 J Bacteriol, 1998
- Cloning and sequencing of the Haemophilus influenzae  **exbB**  and  **exbD**  genes.  Ref: 7828935 Gene, 1995
- Rather, FecA and the TonB,  **ExbB**  and  **ExbD**  proteins are involved in induction of the fec transport genes independent of their role in ferric citrate transport across the outer membrane.  Ref: 7729419 EMBO J, 1995
- **ExbB**  and  **ExbD**  each formed two unique cross linked complexes which were not dependent on the presence of TonB, the outer membrane receptor protein FepA, or the other Exb protein.  Ref: 9811664 J Bacteriol, 1998
- Based on sequence similarities, the Bordetella pertussis tonB  **exbB**   **exbD**  locus was identified on a cloned DNA fragment.  Ref: 10722583 Infect Immun, 2000
- In gram negative bacteria, high affinity iron uptake requires the TonB  **ExbB**   **ExbD**  envelope complex to release iron chelates from their specific outer membrane receptors into the periplasm.  Ref: 10722583 Infect Immun, 2000
- The tolQ, tolR, and tolA genes from Pseudomonas aeruginosa PAO were cloned using degenerate oligonucleotide PCR primers designed based on conserved transmembrane regions of Escherichia coli TolQ and TolR and E. coli and Pseudomonas putida  **ExbB**  and  **ExbD** .  Ref: 8955385 J Bacteriol, 1996
- Analysis of sequence alignments of TolQ and TolR homologues from numerous Gram negative bacterial genomes, together with analyses of the different tolQ tolR mutants, revealed that the TM domains of TolQ and TolR present structural and functional homologies not only to  **ExbB**  and  **ExbD**  of the TonB system but also with MotA and MotB of the flagellar motor.  Ref: 11722743 Mol Microbiol, 2001
- The tonB Bb gene was situated upstream of  **exbB**  and  **exbD**  homologues and downstream of a putative Fur regulated promoter.  Ref: 10517598 Microbiology, 1999
- In gram negative organisms, high affinity transport of iron substrates requires energy transduction to specific outer membrane receptors by the TonB  **ExbB**   **ExbD**  complex.  Ref: 12562789 J Bacteriol, 2003
- Group B colicins, ferric siderophores and vitamin B12 are taken up via the TonB  **ExbB**   **ExbD** , group A colicins via the TolA TolQ TolR system.  Ref: 8316079 Mol Microbiol, 1993
- Pasteurella multocida  **exbB** ,  **exbD**  and tonB genes are physically linked but independently transcribed.  Ref: 12044675 FEMS Microbiol Lett, 2002
- Energy coupled transport across the outer membrane of Escherichia coli  **ExbB**  binds  **ExbD**  and TonB in vitro, and leucine 132 in the periplasmic region and aspartate 25 in the transmembrane region are important for  **ExbD**  activity.  Ref: 8631671 J Bacteriol, 1996
- Neisseria meningitidis tonB,  **exbB** , and  **exbD**  genes Ton dependent utilization of protein bound iron in Neisseriae.  Ref: 9006036 J Bacteriol, 1997
- Cross linking analysis of  **ExbB**  and  **ExbD**  derived size variants demonstrated instead that these  **ExbB**  and  **ExbD**  complexes were homodimers and homotrimers and suggested that  **ExbB**  also interacted with an unidentified protein s.  Ref: 9811664 J Bacteriol, 1998
- Only infection by phage T5 occurs independently of TonB,  **ExbB**  and  **ExbD** .  Ref: 8039674 FEMS Microbiol Lett, 1994
- Reverse transcription RT PCR, using RNA from organisms grown under iron replete and iron restricted conditions, revealed that tonB,  **exbB** ,  **exbD** , tbpB and tbpA are transcribed as a single unit with expression being up regulated in response to iron restriction.  Ref: 12742718 Vet Microbiol, 2003
- Active transport of Fe3 as ferrichrome complex through the outer membrane of Escherichia coli is mediated by the FhuA outer membrane protein and the TonB  **ExbB**   **ExbD**  protein complex in the cytoplasmic membrane.  Ref: 7515827 FEBS Lett, 1994
- Cross linking analysis of epitope tagged  **ExbB**  and  **ExbD**  proteins with TonB antisera afforded detection of a previously unrecognized TonB  **ExbD**  cross linked complex and confirmed the composition of the TonB  **ExbB**  cross linked complex.  Ref: 9811664 J Bacteriol, 1998
- group B colicins use the proteins TonB,  **ExbB** , and  **ExbD** .  Ref: 7545150 J Bacteriol, 1995
- The Ton system consisting of the proteins TonB,  **ExbB**  and  **ExbD**  is required for colicin and microcin import.  Ref: 12423780 Biochimie< MedlineTA>Biochimie, 1995
- Evolutionary relationship of uptake systems for biopolymers in Escherichia coli cross complementation between the TonB  **ExbB**   **ExbD**  and the TolA TolQ TolR proteins.  Ref: 8316079 Mol Microbiol, 1993
- It is proposed that the TonB  **ExbB**   **ExbD**  complex opens via an energized conformation of the TonB protein channels in the outer membrane, formed by proteins which serves as highly specific binding sites for the various ferric siderophores and vitamin B12.  Ref: 7654405 FEMS Microbiol Rev, 1995
- lacZ promoter fusions confirmed that expression of the tonB2  **exbB**   **exbD**  genes is increased under conditions of iron limitation.  Ref: 10689178 FEMS Microbiol Lett, 2000
- Transport of heme by the ShuA protein requires TonB and its accessory proteins  **ExbB**  and  **ExbD** .  Ref: 9393841 Infect Immun, 1997
- Vibrio cholerae iron transport haem transport genes are linked to one of two sets of tonB,  **exbB** ,  **exbD**  genes.  Ref: 9781885 Mol Microbiol, 1998
- It is postulated that FecA, TonB,  **ExbB**  and  **ExbD**  transfer the signal across the outer membrane, while the regulatory protein FecR transmits the signal across the cytoplasmic membrane to FecI in the cytoplasm.  Ref: 7729419 EMBO J, 1995
- Since no energy source is known in the outer membrane it is assumed that energy is provided through the action of the TonB,  **ExbB**  and  **ExbD**  proteins, which are anchored to the cytoplasmic membrane.  Ref: 7688295 EMBO J, 1993
- In addition, outer membrane receptors together with the TonB  **ExbB**   **ExbD**  complex are directly involved in induction of the transcription of ferric citrate and pseudobactin transport genes of Escherichia coli and Pseudomonas putida, respectively.  Ref: 7654405 FEMS Microbiol Rev, 1995
- Specifically, both  **ExbB**  and  **ExbD**  were found to be required for TonB to conformationally respond to proton motive force.  Ref: 12193634 J Bacteriol, 2002
- **ExbB**  and  **ExbD**  proteins are part of the TonB dependent energy transduction system and are encoded by the exb operon in Escherichia coli.  Ref: 12193634 J Bacteriol, 2002
- , Triton X 100 TX 100 did not require the TonB,  **ExbB** , or  **ExbD**  protein.  Ref: 11796379 Antimicrob Agents Chemother, 2002
- Thus, the sequence comparison identified the ORF1, ORF2 and ORF3 as gonococcal homologues of the E. coli tonB,  **exbB**  and  **exbD**  genes.  Ref: 9140974 Mol Microbiol, 1997
- The energy of the proton motive force is postulated to be transduced to the transport proteins by a protein complex that consists of the TonB,  **ExbB** , and  **ExbD**  proteins.  Ref: 11566987 J Bacteriol, 2001
- The electrochemical potential of the cytoplasmic membrane CM energizes transport across the outer membrane, which requires an energy transducing device, consisting of the proteins TonB,  **ExbB**  and  **ExbD** , from the CM to the OM.  Ref: 9377472 Biol Chem, 1997
- Both  **ExbB**  and  **ExbD**  were also required for association of TonB with the cytoplasmic membrane.  Ref: 12193634 J Bacteriol, 2002
- Other biochemical evidence also shows that the proteins that provide energy for iron transport at the outer membrane, such as the TonB  **ExbB**   **ExbD**  system, are structurally very similar across bacterial species.  Ref: 11895294 Curr Top Med Chem, 2001
- Evidence that the absence of  **ExbB**  or  **ExbD**  differentially affected the half lives of newly synthesized and steady state TonB was obtained.  Ref: 7642501 J Bacteriol, 1995
- Quantification of known components of the Escherichia coli TonB energy transduction system TonB,  **ExbB** ,  **ExbD**  and FepA.  Ref: 11967085 Mol Microbiol, 2002
- The activity of the FhuA receptor in the outer membrane of Escherichia coli is dependent on the TonB,  **ExbB**  and  **ExbD**  proteins which are anchored to the cytoplasmic membrane.  Ref: 8039674 FEMS Microbiol Lett, 1994
- Analysis of the nucleotide sequence in the complementing DNA showed that it codes for three genes in the order of  **exbB**   **exbD**  tonB.  Ref: 9074497 Gene, 1997
- Excision of segments as small as 12 amino acids in the largest surface loop of FhuA converted FhuA into an open channel through which ferrichrome and antibiotics diffused independent of TonB  **ExbB**   **ExbD** .  Ref: 7515827 FEBS Lett, 1994
- Cloning and characterization of the  **exbB**   **exbD**  tonB locus of Pasteurella haemolytica A1.  Ref: 9074497 Gene, 1997
- Characterization of the exbBD operon of Escherichia coli and the role of  **ExbB**  and  **ExbD**  in TonB function and stability.  Ref: 7642501 J Bacteriol, 1995
- Transport of bound chelates across the outer membrane depends upon TonB  **ExbB**   **ExbD** , a cytoplasmic membrane localized complex that transduces energy from the proton motive force to high affinity receptors in the outer membrane.  Ref: 9643536 Mol Microbiol, 1998
- Escherichia coli and related Gram negative bacteria contain an energy coupled transport system through the outer membrane which consists of the proteins TonB,  **ExbB** ,  **ExbD**  anchored in the cytoplasmic membrane and receptors in the outer membrane.  Ref: 8316089 Mol Microbiol, 1993
- The reconstitution in Escherichia coli of the S. marcescens Has system demonstrated that haem uptake is dependent on the E. coli  **ExbB** ,  **ExbD**  and TonB proteins.  Ref: 11737642 Mol Microbiol, 2001
- Following incubation on media containing hemoglobin, N. meningitidis IR3436 tonB  **exbB**   **exbD**  deletion mutant and N. gonorrhoeae PD3401 tonB insertional mutant give rise to colonies which can grow with hemoglobin.  Ref: 10986265 J Bacteriol, 2000
- A complex of three membrane spanning proteins TonB,  **ExbB**  and  **ExbD**  couples the chemiosmotic potential of the cytoplasmic membrane with siderophore uptake across the outer membrane.  Ref: 12409204 Biochim Biophys Acta, 2002
- A putative Fur binding sequence located upstream from tonB was detected in a Fur titration assay, indicating that the tonB  **exbB**   **exbD**  operon may be Fur repressed in high iron growth conditions.  Ref: 10722583 Infect Immun, 2000
- Energy from the cytoplasmic membrane is transferred to the outer membrane with the aid of the Ton system, consisting of the proteins TonB,  **ExbB** , and  **ExbD** .  Ref: 8631671 J Bacteriol, 1996
- These results were consistent with the idea that  **ExbB**  and  **ExbD**  play roles in the assembly of TonB into an energy transducing complex.  Ref: 7642501 J Bacteriol, 1995
- The ratio of these proteins was calculated at one TonB two  **ExbD**  seven  **ExbB**  under all four growth conditions tested.  Ref: 11967085 Mol Microbiol, 2002
- TolQ TolR interacted through TonB, and  **ExbB**   **ExbD**  interacted through TolA with the outer membrane receptors and colicins.  Ref: 8316079 Mol Microbiol, 1993
- Sequencing of the Ton system genes revealed that the arrangement of the genes was  **exbB**   **exbD**  tonB.  Ref: 9423852 Infect Immun, 1998
- Downstream of comA two partially overlapping open reading frames termed  **exbB**  and  **exbD**  were found coding for putative proteins similar to proteins required for macromolecule uptake in Escherichia coli and present in other Gram negative bacteria.  Ref: 11446513 Res Microbiol, 2001
- The  **exbB** ,  **exbD**  and tonB genes of the Pasteurella multocida animal pathogen have been cloned by complementation of an Escherichia coli tonB mutant.  Ref: 12044675 FEMS Microbiol Lett, 2002
- Crystal structures of two outer membrane transport proteins, FhuA and FepA, and biochemical data reveal strong long range conformational changes of the proteins upon binding of Fe 3 siderophore complexes and in response to energy transfer from the cytoplasmic membrane into the outer membrane via the TonB  **ExbB**   **ExbD**  protein complex.  Ref: 11934617 Curr Opin Microbiol, 2002
- However, inducible levels of TX 100 resistance in gonococci had an absolute requirement for the TonB  **ExbB**   **ExbD**  system, suggesting that such resistance in gonococci has an energy requirement above and beyond that required for constitutive pump activity.  Ref: 11796379 Antimicrob Agents Chemother, 2002
- TonB, the energy transducer, appears to go through a cycle during energy transduction, with the absence of both  **ExbB**  and  **ExbD**  creating blocks at two points i in the inability of TonB to respond to the cytoplasmic membrane proton motive force and ii in the conversion of TonB from a high affinity outer membrane association to a high affinity cytoplasmic membrane association.  Ref: 12193634 J Bacteriol, 2002
- PCR approaches allowed the amplification, cloning and sequencing of A. suis tonB,  **exbB** ,  **exbD** , tbpB and tbpA homologues.  Ref: 12742718 Vet Microbiol, 2003
- In the absence of  **ExbB**  or  **ExbD** , newly synthesized TonB was degraded with a half life of 5 to 10 min, while the half life of TonB under steady state conditions was significantly longer, approximately 30 min.  Ref: 7642501 J Bacteriol, 1995
- In Gram negative bacteria, the TonB  **ExbB**   **ExbD**  inner membrane multiprotein complex is required for active transport of diverse molecules through the outer membrane.  Ref: 11737642 Mol Microbiol, 2001
- In this study, we report the per cell numbers of TonB,  **ExbB** ,  **ExbD**  and FepA for cells grown under iron replete and iron limited conditions.  Ref: 11967085 Mol Microbiol, 2002
- In this study, in vivo formaldehyde cross linking analysis was used to investigate the interactions of T7 epitope tagged  **ExbB**  or  **ExbD**  proteins.  Ref: 9811664 J Bacteriol, 1998
- Allelic replacement of the wild type H. ducreyi  **exbB** ,  **exbD** , and tonB loci with the  **exbB** ,  **exbD** , and tonB deletion resulted in an H. ducreyi isogenic mutant unable to utilize hemoglobin but able to utilize hemin at the same levels as the parent strain to fulfill its heme requirement.  Ref: 9423852 Infect Immun, 1998
- Energy flows from the cytoplasmic into the outer membrane via a complex consisting of the TonB,  **ExbB**  and  **ExbD**  proteins which are anchored in the cytoplasmic membrane.  Ref: 7654405 FEMS Microbiol Rev, 1995
- Nucleotide sequence analysis of DNA fragments subcloned from the Hb complementing cosmids identified four open reading frames, three of them homologous to Pseudomonas putida, E. coli, and Haemophilus influenzae  **exbB** ,  **exbD** , and tonB genes.  Ref: 9006036 J Bacteriol, 1997
- They share homology with the  **ExbB** ,  **ExbD** , and TonB proteins, respectively.  Ref: 11418549 J Bacteriol, 2001
- This genetic organization has been reported in Haemophilus influenzae, H. ducreyi, Pseudomonas putida and Vibrio cholerae, and may represent a separate lineage of evolution from that of the Enterobacteriaceae in which tonB is unlinked with the accessory genes  **exbB**  and  **exbD** .  Ref: 9074497 Gene, 1997
- The signal elicited by ferric citrate crosses the outer membrane via TonB,  **ExbB** , and  **ExbD** .  Ref: 8602163 Mol Gen Genet, 1996
- The energy is transduced to FecA by the inner membrane complex, TonB,  **ExbB** , and  **ExbD** .  Ref: 12618451 J Bacteriol, 2003
- A second tonB gene in Pseudomonas aeruginosa is linked to the  **exbB**  and  **exbD**  genes.  Ref: 10689178 FEMS Microbiol Lett, 2000
- The  **exbB**  and  **exbD**  genes of Haemophilus influenzae Hi were cloned and sequenced.  Ref: 7828935 Gene, 1995
- It is thought that the proteins TonB,  **ExbB** , and  **ExbD**  function as a coupling device between the cytoplasmic membrane and the outer membrane.  Ref: 9457864 J Bacteriol, 1998
- In contrast, E. coli tonB G174R, V178I was sensitive to colicin Ia and resistant to colicin B unless TonB,  **ExbB**  and  **ExbD**  were overproduced which resulted in colicin B sensitivity.  Ref: 8039672 FEMS Microbiol Lett, 1994
- Inducible, but not constitutive, resistance of gonococci to hydrophobic agents due to the MtrC MtrD MtrE efflux pump requires TonB  **ExbB**   **ExbD**  proteins.  Ref: 11796379 Antimicrob Agents Chemother, 2002
- **ExbB**  and  **ExbD**  do not function independently in TonB dependent energy transduction.  Ref: 12193634 J Bacteriol, 2002
- Vibrio cholerae was found to have two sets of genes encoding TonB,  **ExbB**  and  **ExbD**  proteins.  Ref: 9781885 Mol Microbiol, 1998
- In this study, Escherichia coli TonB was found to be distributed in sucrose density gradients approximately equally between the cytoplasmic membrane and the outer membrane fractions, while two proteins with which it is known to interact,  **ExbB**  and  **ExbD** , as well as the NADH oxidase activity characteristic of the cytoplasmic membrane, were localized in the cytoplasmic membrane fraction.  Ref: 9159515 Mol Microbiol, 1997
- When  **ExbB**  and  **ExbD** , as well as their cross talk competent homologues ToIQ and ToIR, were absent, TonB was found associated with the outer membrane.  Ref: 9159515 Mol Microbiol, 1997
- TonB,  **ExbB**  and  **ExbD**  are only required for outer membrane transport.  Ref: 7688295 EMBO J, 1993
- Energy coupled transport and signal transduction through the gram negative outer membrane via TonB  **ExbB**   **ExbD**  dependent receptor proteins.  Ref: 7654405 FEMS Microbiol Rev, 1995
- The last is involved in energy transduction between the inner and the outer membrane, and its conformation has been shown to depend on the presence of the proton motive force PMF,  **ExbB** , and  **ExbD** .  Ref: 11418549 J Bacteriol, 2001
- Cross complementation between the two systems was demonstrated in that tolQ tolR mutants transformed with plasmids carrying  **exbB**   **exbD**  became sensitive to group A colicins, and  **exbB**   **exbD**  mutants transformed with plasmid encoded tolQ tolR became sensitive to group B colicins.  Ref: 8316079 Mol Microbiol, 1993
